# Imaging of Reactive Astrogliosis by Positron Emission Tomography

**DOI:** 10.3389/fnins.2022.807435

**Published:** 2022-02-08

**Authors:** Ryuichi Harada, Shozo Furumoto, Yukitsuka Kudo, Kazuhiko Yanai, Victor L. Villemagne, Nobuyuki Okamura

**Affiliations:** ^1^Department of Pharmacology, Tohoku University Graduate School of Medicine, Sendai, Japan; ^2^Cyclotron and Radioisotope Center, Tohoku University, Sendai, Japan; ^3^Department of New Therapeutics Innovation for Alzheimer’s and Dementia, Institute of Development and Aging, Tohoku University, Sendai, Japan; ^4^Department of Molecular Imaging and Therapy, Austin Health, Melbourne, VIC, Australia; ^5^Department of Psychiatry, University of Pittsburgh, Pittsburgh, PA, United States; ^6^Division of Pharmacology, Faculty of Medicine, Tohoku Medical and Pharmaceutical University, Sendai, Japan

**Keywords:** reactive astrogliosis, MAO-B, imidazoline_2_ binding site, PET, radiotracers

## Abstract

Many neurodegenerative diseases are neuropathologically characterized by neuronal loss, gliosis, and the deposition of misfolded proteins such as β-amyloid (Aβ) plaques and tau tangles in Alzheimer’s disease (AD). In postmortem AD brains, reactive astrocytes and activated microglia are observed surrounding Aβ plaques and tau tangles. These activated glial cells secrete pro-inflammatory cytokines and reactive oxygen species, which may contribute to neurodegeneration. Therefore, *in vivo* imaging of glial response by positron emission tomography (PET) combined with Aβ and tau PET would provide new insights to better understand the disease process, as well as aid in the differential diagnosis, and monitoring glial response disease-specific therapeutics. There are two promising targets proposed for imaging reactive astrogliosis: monoamine oxidase-B (MAO-B) and imidazoline_2_ binding site (I_2_BS), which are predominantly expressed in the mitochondrial membranes of astrocytes and are upregulated in various neurodegenerative conditions. PET tracers targeting these two MAO-B and I_2_BS have been evaluated in humans. [^18^F]THK-5351, which was originally designed to target tau aggregates in AD, showed high affinity for MAO-B and clearly visualized reactive astrocytes in progressive supranuclear palsy (PSP). However, the lack of selectivity of [^18^F]THK-5351 binding to both MAO-B and tau, severely limits its clinical utility as a biomarker. Recently, [^18^F]SMBT-1 was developed as a selective and reversible MAO-B PET tracer via compound optimization of [^18^F]THK-5351. In this review, we summarize the strategy underlying molecular imaging of reactive astrogliosis and clinical studies using MAO-B and I_2_BS PET tracers.

## Introduction

Alzheimer’s disease (AD) is neuropathologically characterized by neuronal loss, deposition of β-amyloid (Aβ) plaques, and neurofibrillary tangles (NFT) (Jellinger and Bancher, 1998). Astrogliosis and microgliosis have been observed surrounding both dense-core Aβ plaques and NFT (Alibhai et al., 2018). Neuroinflammatory changes, characterized by reactive astrocytes and activated microglia, are considered to be a secondary process following Aβ and tau accumulation and contribute greatly to neurodegeneration throughout the course of AD (Ingelsson et al., 2004). Astrocytes are the most abundant glial cells in the brain, which are involved in a wide range of physiological functions including synaptic plasticity, the formation of astroglio-vascular units, ion homeostasis, regulation of gliotransmitters such as glutamate and γ-aminobutyric acid (GABA), water transport, and regulation of local blood flow. When astrocytes respond to pathological conditions including traumatic brain injury, ischemia, infection, and misfolded protein accumulation, they exhibit morphological and phenotypic changes to transform into reactive astrocytes (Pekny et al., 2016). A recent consensus paper proposed that reactive astrogliosis should be defined as the spectrum of molecular, morphological, and functional changes in response to pathological conditions (Escartin et al., 2021). Reactive astrocytes display changes in gene expression and overexpress intermediate filaments such as glial fibrillary acid protein (GFAP), vimentin, and nestin. Reactive astrocytes secrete inflammatory mediators such as cytokines [interleukin-1β (IL-1β) and IL-6] and tumor necrosis factor-α (TNF-α), leading to the inflammatory, as observed in AD (Carter et al., 2019b). Reactive astrocytes have also been classified as neurotoxic (A1) and neuroprotective (A2) phenotypes. A1 astrocytes, observed in various neurodegenerative diseases, release the neurotoxic complement C3d and induce neuronal death. A2 astrocytes, commonly observed in ischemia, promote neuronal survival (Liddelow et al., 2017). However, this binary classification does not truly reflect the wide spectrum of molecular, morphological, and functional changes observed in reactive astrogliosis. The term “astrogliosis” refers to a spectrum of potentially protective or deleterious pathways underlying complex phenotypic and functional changes reflecting both a loss of normal function and a gain of toxic function that can be region specific and associated with specific disease stages (Escartin et al., 2021). Therefore, a true classification is not possible given the variety of functions and morphologies, better served by the sum effect being preponderant protective or preponderant toxic (Escartin et al., 2021). Reactive astrogliosis is observed not only in AD, but also in various neurodegenerative diseases including amyotrophic lateral sclerosis (ALS), frontotemporal lobar degeneration (FTLD), Creutzfeldt-Jakob disease (CJD), progressive supranuclear palsy (PSP), Parkinson’s disease (PD), multiple system atrophy (MSA), and chronic traumatic encephalopathy (CTE), as well as in other neurological conditions such as epilepsy and stroke. Therefore, *in vivo* imaging of reactive astrocytes by positron emission tomography (PET) would provide new insights and a better understanding of the underlying disease process, aid in the differential diagnosis, and monitoring the glial response of disease-modifying therapeutics. In this review, we describe the recent advances in the development of PET tracers for imaging reactive astrocytes and the clinical applications of these new tracers.

## Molecular Targets in the Development of Positron Emission Tomography Tracer for Imaging Astrogliosis

For imaging astrogliosis in the brain, translocator protein 18-kDa (TSPO) has been considered as a target in the development of PET tracers (Escartin et al., 2021), although it is also widely recognized as an imaging marker for activated microglia. Since TSPO is overexpressed in both activated microglia and reactive astrocytes, there are technical limitations in achieving high glial cell-type specificity of PET tracers using TSPO as a molecular target. Furthermore, it is difficult to differentiate between neuroprotective and neurotoxic immune responses (Jain et al., 2020; Zhang et al., 2021). TSPO expression in AD and control brains was recently characterized in detail from postmortem brain samples (Gui et al., 2020). The findings of this study are as follows: (1) TSPO is expressed not only in microglia but also in astrocytes, endothelial cells, and vascular smooth muscle cells. (2) There is a substantial overlap in TSPO levels between control and AD brains. (3) The TSPO cortical burden did not correlate with the burden of activated microglia and reactive astrocytes. (4) The *TSPO* rs6971 Single Nucleotide Polymorphism (SNP) has been associated with a variable binding of TSPO radioligands, it does not affect neuropathological changes in AD. These findings suggest that a more specific and selective targets beyond TSPO are required for PET imaging of activated glial cells (Boche et al., 2019; Jain et al., 2020).

**FIGURE 1:**
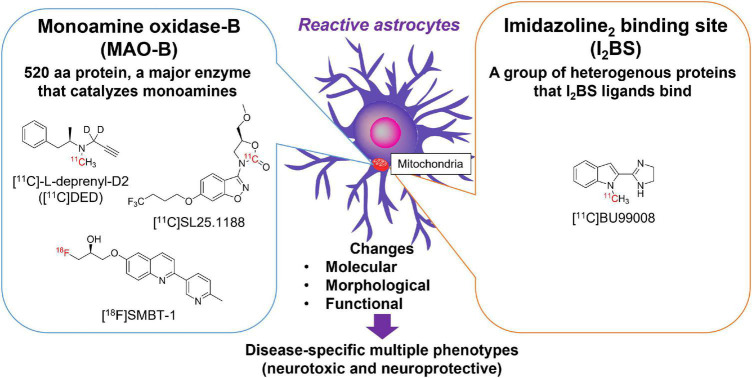
Molecular targets for imaging reactive astrogliosis: monoamine oxidase-B (MAO-B) and imidazoline_2_ binding site (I_2_BS). Chemical structures of selective PET tracers for MAO-B and I_2_BS tested in humans.

Reactive astrocytes are characterized by the overexpression of intermediate filaments such as GFAP, a gold standard marker of reactive astrocytes (Escartin et al., 2021). However, there are no available small molecular compounds for GFAP with high affinity and selectivity. Alternative surrogate targets of astrogliosis are monoamine oxidase-B (MAO-B) and imidazoline_2_ binding sites (I_2_BS), which are overexpressed in the outer mitochondrial membrane of reactive astrocytes (Ekblom et al., 1993; Regunathan et al., 1993; Figure 1). As many therapeutic drug candidates have been developed for pharmacological regulation of MAO-B and I_2_BS, these compounds have been radiolabeled and tested as surrogate imaging probes for reactive astrogliosis (Carter et al., 2019b; Figure 1). MAO-B, a 520 amino acid protein, is the major enzyme that metabolizes monoamines such as dopamine, tyramine, and histamine in the human brain. MAO-B inhibitors such as selegiline (L-deprenyl), rasagiline, and safinamide have been used for the treatment of PD (Youdim et al., 2006). The crystal structures of human MAO-B revealed that irreversible MAO-B inhibitors such as L-deprenyl and rasagiline, which have an *N*-propargyl group covalently attached to the *N*5 atom of the flavin adenine dinucleotide (FAD) cofactor of MAO-B (Binda et al., 2002, 2004), while the reversible inhibitor safinamide binds non-covalently to MAO-B and occupy both the entrance and the substrate cavities (Binda et al., 2007). MAO-B is predominantly expressed in the outer membrane of the mitochondria in astrocytes but also in monoaminergic neurons, while it is much lower concentrations are found in oligodendrocytes, microglia, and endothelial cells (Levitt et al., 1982) (Brainrnaseq.org). Higher levels of MAO-B expression are observed in the basal forebrain, substantia nigra, basal ganglia, thalamus, and hippocampal uncus relative to the cerebellar cortex (Jossan et al., 1991a; Tong et al., 2013). There were significantly positive correlations with age (from 21 h to 99 years) for MAO-B in the frontal cortex (*r* = 0.54) (Tong et al., 2013). Age-related changes could be separated into three phases—within 1 year of age, 1–4 years, and thereafter. After controlling for the effect of age, there was also a significant correlations in adults only (≧18 years, *r* = 0.64), childhood only (<18 years, *r* = 0.61), or infancy only (<1 year, *r* = 0.82). MAO-B levels are significantly correlated with GFAP immunoreactivity in the AD brain (Saura et al., 1994; Jo et al., 2014). Elevation of MAO-B levels in autopsy-confirmed AD brains has been consistently observed in mRNA levels and *in vitro* binding studies with MAO-B radioligands such as [^3^H]-L-deprenyl and [^3^H]lazabemide (Jossan et al., 1991b; Saura et al., 1994; Gulyas et al., 2011; Marutle et al., 2013; Jo et al., 2014; Ni et al., 2021). MAO-B was also upregulated in postmortem brain tissues from ALS, epilepsy, Parkinsonian syndromes such as PSP, PD, and MSA (Aquilonius et al., 1992; Kumlien et al., 1992; Ekblom et al., 1993; Tong et al., 2017).

I_2_BS are an imidazoline binding sites compound of a group of heterogeneous proteins that are preferentially labeled by idazoxan and 2-(Benzofuran-2-yl)-2-imidazoline hydrochloride (2-BFI). The characteristics of imidazoline binding sites has been previously reviewed in detail (Bousquet et al., 2020). I_2_BS are involved in regulation of GFAP expression and MAO-B activity (Olmos et al., 1994). Western blot analysis using polyclonal antiserum against idazoxan-binding proteins revealed four different protein bands (30, 45–57, 66, and 85 kDa) in rats and rabbits (Olmos et al., 1999). Brain creatine kinase was identified using a 2-BFI affinity column, which corresponded to an approximately 45 kDa band (Kimura et al., 2009). I_2_BS are predominantly located at the outer mitochondrial membrane and are thought to be novel allosteric binding sites of MAO-A and MAO-B (Tesson et al., 1991, 1995; Parini et al., 1996). Both of MAO-B and I_2_BS are not only expressed in astrocytes but also in neurons. 2-BFI binds to the tranylcypromine-inhibited-MAO-B form with high affinity, but presents low binding affinity to native human MAO-B. In addition, the crystal structure of the MAO-B-ligand complex demonstrated that 2-BFI binds to a site distinct from the substrate-binding cavity of MAO-B (Bonivento et al., 2010). However, it is likely that proteins other than MAO also express I_2_BS, as substantial specific radioligand binding remained in MAO knockout mice (Remaury et al., 2000; Anderson et al., 2006). The other bands detected by idazoxan remain unknown (Bousquet et al., 2020). [^3^H]idazoxan binding in the human brain is greater in the basal ganglia, such as the caudate nucleus, putamen, substantia nigra, pons, and hippocampus than in the cerebellar cortex (De Vos et al., 1991). The density of I_2_BS determined by [^3^H]idazoxan was ∼35-fold lower than that of the MAO-B binding site determined by [^3^H]Ro 19-6327 (a.k.a. lazabemide). As observed in MAO-B, the density of I_2_BS increases linearly in an age-dependent manner in the frontal cortex during normal aging processes (Sastre and Garcia-Sevilla, 1993). The average increase per decade of I_2_BS was 10.5 fmol/mg of protein (∼16% mean increase per decade between 20 and 70 years), while that of MAO-B was 44.7 fmol/mg of protein (∼51% mean increase per decade between 20 and 70 years). Therefore, age-related changes in brain I_2_BS levels were 4-times lower than with those in MAO-B. Although the regional binding of radiotracers for MAO-B and I_2_BS in the human brain is correlated with each other, the binding sites between MAO-B and I_2_BS radiotracers are distinct (Sastre and Garcia-Sevilla, 1993). The density of [^3^H]idazoxan binding in the AD brain is higher than that in the control brain (Ruiz et al., 1993). This finding is consistent with the results of western blot analysis using anti-imidazoline receptor protein anti-serum (Garcia-Sevilla et al., 1998). A novel PET tracer [^11^C]BU99008, was developed as an I_2_BS ligand. BU99008 possesses high affinity (*K*_i_ = 1.4 nM, *K*_*D*_ = 1.3 nM) and selectivity to I_2_BS, compared with α_2_-aderenoceptor in rat brain (*K*_i_ = 1,273 nM) (Tyacke et al., 2012). Recently, BU99008 was established as an astroglial marker by analyzing postmortem AD brains (Kumar et al., 2021). The spatial distribution of [^3^H]BU99008 was similar to that of the MAO-B radioligand [^3^H]-L-deprenyl in large frozen sections from the control and AD groups. However, there was no binding competition, suggesting that these two radioligands have different binding sites.

## Clinical Studies Using Positron Emission Tomography Radiotracers for Monoamine Oxidase-B and Imidazoline_2_ Binding Site

### Monoamine Oxidase-B: [^11^C]-L-Deprenyl and [^11^C]DED

Several PET tracers with high binding affinity to MAO-B or I_2_BS have been investigated for the *in vivo* visualization of reactive astrogliosis. The first clinical study of MAO-B PET imaging was conducted using [^11^C]-L-deprenyl, a selective and irreversible inhibitor of MAO-B (Fowler et al., 1987). However, [^11^C]-L-deprenyl showed a rapid rate of first pass extraction relative to transport, resulting in a reduction in the sensitivity of the tracer in regions of high MAO-B concentrations, including the basal ganglia and thalamus on top of being influenced by blood flow. To minimize these effects, [^11^C]-L-deprenyl D2 ([^11^C]DED) was developed to improve tracer sensitivity (Fowler et al., 1995). [^11^C]DED successfully reduced the first pass extraction of the tracer and improved sensitivity. There was a good correlation between MAO-B concentration ratios (ROI-to-cerebellum) and slope (*K*_i_) ratios, which was estimated from graphical analysis for irreversible tracers. To date, [^11^C]DED is the most widely used tracer for MAO-B PET in clinical studies to access MAO-B *in vivo* (Engler et al., 2012; Fowler et al., 2015; Rodriguez-Vieitez et al., 2016). MAO-B levels measured by [^11^C]DED increased linearly in an age-dependent manner in whole brain regions during the normal aging process except for the cingulate gyrus (Fowler et al., 1997), consistent with postmortem data (Tong et al., 2013). Elevated [^11^C]DED binding has been observed in several neurodegenerative conditions including traumatic brain injury (TBI), AD, ALS, and CJD (Fowler et al., 1999; Johansson et al., 2007; Santillo et al., 2011; Engler et al., 2012). However, another group reported that [^11^C]DED binding was not elevated in patients with dementia, but was increased in PiB positive mild cognitive impairment (MCI) subjects (prodromal AD) (Carter et al., 2012). No regional correlations were found between [^11^C]PiB, [^11^C]DED, and [^18^F]FDG. On the other hands, some TBI patients showed an elevation of [^11^C]DED in the regions corresponding to glucose hypometabolism (Fowler et al., 1999). [^11^C]DED binding in the parahippocampus was negatively correlated with gray matter density in prodromal AD (Choo et al., 2014). They also reported that [^11^C]DED binding was positively correlated with [^11^C]PiB retention in the parahippocampus of AD. This region was also reported to significantly increase [^11^C]PiB retention in AD dementia than prodromal AD (Nordberg et al., 2013). Furthermore, significant [^11^C]DED binding was observed in pre-symptomatic mutation carriers of autosomal dominant AD (ADAD) that showed cerebral glucose metabolism were largely preserved (Scholl et al., 2015). Longitudinal studies in ADAD demonstrated the positive rates of change in [^11^C]PiB retention and negative rates of changes in [^11^C]DED binding and [^18^F]FDG uptake in mutation carriers (Rodriguez-Vieitez et al., 2016). In addition, it showed high variability of [^11^C]DED binding in mutation carriers and non-carriers (Rodriguez-Vieitez et al., 2016). Longitudinal reduction in [^11^C]DED binding was also correlated with progressive cerebral glucose hypometabolism in pre-symptomatic mutation carriers (Carter et al., 2019a). Although it is considered that reduction of [^11^C]DED binding is probably due to astrocytic dysfunction and atrophy by a downstream effect of the early MAO-B upregulation and a reflection of chronic neuroinflammation (Carter et al., 2019a), it remains unclear because many postmortem studies demonstrated the elevation of MAO-B levels in AD (Jossan et al., 1991b; Saura et al., 1994; Gulyas et al., 2011; Marutle et al., 2013; Ni et al., 2021). Furthermore, [^11^C]DED presents difficulties in tracer binding quantification due to its irreversible binding kinetics and the presence of blood-brain barrier (BBB) penetrating radiolabeled metabolites including methamphetamine and amphetamine (Fowler et al., 1988; Azzaro et al., 2007; Narayanaswami et al., 2019), which bind to the monoamine transporter in the brain.

### Monoamine Oxidase-B: [^11^C]SL25.1188

As an alternative radiotracer, [^11^C]SL25.1188 was developed as a MAO-B PET tracer and has shown more favorable reversible pharmacokinetics without BBB permeable radiolabeled metabolites in humans (Saba et al., 2010; Rusjan et al., 2014). A clinical study demonstrated that regional total distribution volume was highly correlated with previous reported MAO-B concentration using port-mortem brain samples, but the lack of *in vivo* blocking study with selective MAO-B inhibitors in humans. Assessment of MAO-B density was investigated in patients with major depressive disorder because serotonergic neurons also contain MAO-B. [^11^C]SL25.1188 PET demonstrated greater MAO-B density in the prefrontal cortex of patients with major depressive disorder (Moriguchi et al., 2019). Very recently, a [^11^C]SL25.1188 PET study has been reported that trending lower tracer uptake in posttraumatic stress disorder (PTSD), which is a debilitating mental health condition that results from exposure to traumatic events (Gill et al., 2021). Although [^11^C]SL25.1188 looks a promising MAO-B PET tracer, it was mainly used in the studies focusing on psychiatric disorders and there are few reported clinical studies in neurodegenerative diseases.

### Tau and Monoamine Oxidase-B: [^18^F]THK-5351

**FIGURE 2:**
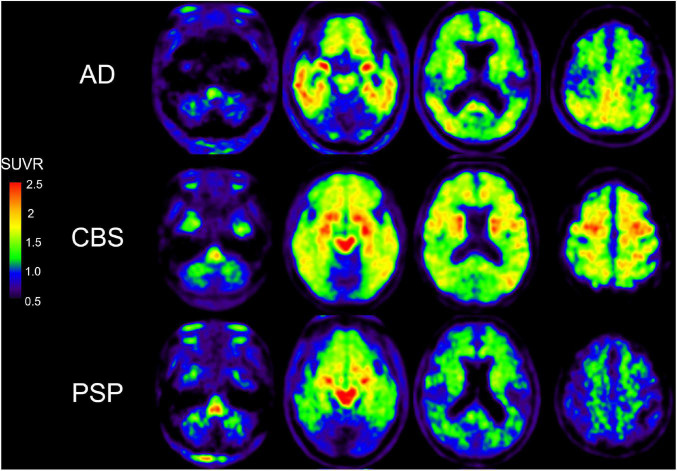
[^18^F]THK-5351 PET images in AD, CBS, and PSP subjects. [^18^F]THK-5351 retention in the precentral gyrus clearly can distinguish CBS from PSP and AD. [^18^F]THK-5351 retention in the midbrain distinguished PSP and CBS from AD (Okamura et al., 2018).

Although a 2-arylquinoline derivative [^18^F]THK-5351 was originally developed as a tau PET ligand (Harada et al., 2016; Tago et al., 2016), validation studies revealed off-target binding of [^18^F]THK-5351 to MAO-B (Ng et al., 2017; Harada et al., 2018). [^18^F]THK-5351 binds to MAO-B with high affinity and the *in vivo* [^18^F]THK-5351 binding was correlated with MAO-B levels in autopsy-confirmed AD and PSP patients (Harada et al., 2018; Ishiki et al., 2018). In addition, *in vivo* blocking studies with oral selegiline and rasagiline revealed specific binding of [^18^F]THK-5351 to MAO-B in AD and PSP brains (Ng et al., 2017, 2019), where [^18^F]THK-5351 binding was reduced on average by 37–52% in the post-selegiline scans and 53–89% in the post-rasagiline scans (Ng et al., 2017, 2019). These results suggested [^18^F]THK-5351 would be useful for the evaluation of reactive astrogliosis and several researchers examined the potential clinical utility of [^18^F]THK-5351 in various disease conditions including AD and non-AD conditions. Although there were a lot of limitations to interpret [^18^F]THK-5351 PET images on the biological point of view due to the lack of selectivity, many clinical studies using [^18^F]THK-5351 imply the association of reactive astrogliosis in various conditions. Clinical PET studies demonstrated high and different distribution of [^18^F]THK-5351 retention in sites susceptible to tau deposition in AD and non-AD conditions such as Parkinsonian syndromes (Harada et al., 2016; Kikuchi et al., 2016; Brendel et al., 2017; Ishiki et al., 2017; Shimizu et al., 2018; Schonecker et al., 2019; Hsu et al., 2020; Ezura et al., 2021; Figure 2). Furthermore, [^18^F]THK-5351 retention mirrors glucose hypometabolism measured with [^18^F]fluorodeoxyglucose in the focal variants of AD (Kang et al., 2017; Park et al., 2021). [^18^F]THK-5351 PET studies in AD also showed different tracer retention in three distinct subtypes of AD: medial temporal dominant subtype, parietal-dominant subtype, and diffuse atrophy subtype (Jeon et al., 2019). Semantic variant primary progressive aphasia (svPPA) is typically associated with frontotemporal lobar degeneration with the accumulation of TDP-43 (FTLD-TDP type C), and is not commonly due to primary tauopathy or AD (Grossman, 2010). In svPPA patients, [^18^F]THK-5351 demonstrated that elevated retention in the anteroinferior and lateral temporal cortices compared with normal control, and in the left inferior and temporal polar region compared with AD (Kobayashi et al., 2018; Lee et al., 2018; Schaeverbeke et al., 2018; Takaya et al., 2018; Son et al., 2019). [^18^F]THK-5351 showed remarkable retention in ALS and multiple sclerosis (Ishibashi et al., 2020; Higashihara et al., 2021; Saitoh et al., 2021). As observed in [^11^C]DED PET study (Engler et al., 2012), [^18^F]THK-5351 retention was elevated in autopsy-confirmed sporadic CJD patient (Kim et al., 2019). Neuropathological examination of this case showed abundant MAO-B expressing reactive astrogliosis and no remarkable tau deposition. These reports indicate that [^18^F]THK-5351 PET can detect overexpressed MAO-B in reactive astrocytes *in vivo*. [^18^F]THK-5351 PET has also been applied for clinical assessment in non-neurodegenerative diseases. In a patient who suffered a middle cerebral artery infarction, PET image showed intense [^18^F]THK-5351 retention along the ipsilateral pyramidal tract, likely reflecting the gliosis accompanying Wallerian degeneration (Ishibashi et al., 2017). [^18^F]THK-5351 retention in ischemic stroke patients was elevated in peri-stroke areas, but most importantly, in areas remote from the stroke lesion, suggesting that [^18^F]THK-5351 PET reflects gliosis associated with widespread ischemia-related and associated with microstructural disruption (Huang et al., 2020). Prominent [^18^F]THK-5351 retention was also reported in a patient after traumatic brain injuries (Uchida et al., 2021) and in a patient with glioblastoma and associated gliosis (Tago et al., 2019; Mitamura et al., 2021).

### Imidazoline_2_ Binding Site: [^11^C]BU99008

Compared to MAO-B PET tracer, only [^11^C]BU99008 was tested in humans as a PET tracer for imaging I_2_BS. BU99008 was labeled with carbon-11 and investigated using PET (Kealey et al., 2013). [^11^C]BU99008 showed a robust brain entry after intravenous administration and high retention in the basal ganglia, moderate in the cortex, and lowest in the cerebellum in porcine, rhesus monkey, and humans (Parker et al., 2014; Tyacke et al., 2018). These findings were consistent with the reported *in vitro* distribution of I_2_BS in the brain. The *in vivo* binding of [^11^C]BU99008 was blocked by pretreatment with idazoxan, but not with the non-selective MAO inhibitor, isocarboxazid, suggesting high selectivity of this tracer to I_2_BS in humans (Tyacke et al., 2018). After testing human biodistribution and radiation dosimetry of [^11^C]BU99008 (Venkataraman et al., 2018), clinical studies were expanded to the patients with neurodegenerative diseases. Early PD patients showed an elevation of [^11^C]BU99008 in frontal, temporal, parietal, and occipital cortical regions, while moderate/advanced PD patients showed a reduction of [^11^C]BU99008 uptake across the whole brain (Wilson et al., 2019). A recent [^11^C]BU99008 PET study in AD demonstrated higher tracer uptake in frontal, temporal, occipital, and medial temporal regions of Aβ-positive cognitively impaired subjects. There was a positive correlation between [^11^C]BU99008 and [^18^F]Florbetaben binding in cognitively impaired subjects (Calsolaro et al., 2021). Since there are a few reports about [^11^C]BU99008 clinical PET studies, further studies are required.

### Comparison of Monoamine Oxidase-B and Imidazoline_2_ Binding Site Positron Emission Tomography Tracers

Comparison of PET tracers for imaging MAO-B and I_2_BS tested in humans were shown in Table 1. Although there are several clinically available MAO-B PET tracers and single I_2_BS PET tracer, the performance of each tracers remains unknown in various conditions. A direct head-to-head comparison study between [^11^C]-L-deprenyl (prototype of [^11^C]DED) and [^18^F]THK-5351 in patients with non-AD neurological disorders whose brains are not expected to harbor tau pathology concluded that [^18^F]THK-5351 was superior to [^11^C]-L-deprenyl for visualizing lesions undergoing reactive astrogliosis (Ishibashi et al., 2021). [^18^F]THK-5351 PET (*BP*_ND_ and SUV) images clearly identified the affected regions undergoing astrogliosis in patients with cerebral infarction, progressive multifocal leukoencephalopathy, and multiple sclerosis. Although [^11^C]-L-deprenyl *K*_i_/*k*_1_ maps were similar to [^18^F]THK-5351 PET images, the signal-to-noise ratio of [^11^C]-L-deprenyl PET images was much lower than that of [^18^F]THK-5351 PET images. *K*_i_ maps and SUV images of [^11^C]-L-deprenyl seemed to be affected by blood flow due to its irreversible binding properties as described above. [^11^C]-L-deprenyl shows irreversible binding kinetics, while [^11^C]SL25.1188 shows reversible binding kinetics as like [^18^F]THK-5351. Future head-to-head comparison studies are required to characterize the performance of reversible MAO-B PET tracers. In addition, head-to-head studies between I_2_BS and MAO-B PET tracers to compare degree of tracer binding as well as regional distribution of the tracers are also certainly warranted. Considering the non-selective binding properties of [^18^F]THK-5351 to MAO-B and AD-tau, [^18^F]THK-5351 is likely to be useful in non-AD patients whose brains are not expected to harbor tau pathology. However, it is important to ensure the specificity and sensitivity of MAO-B PET tracers because astrocyte dysfunction occurs at the early disease stages across the AD spectrum (Leclerc and Abulrob, 2013).

**TABLE 1:** Comparison among MAO-B and I_2_BS PET tracers under various conditions in clinical studies.

Tracers	Targets	Binding kinetics	Tracer uptake (Compared with age-matched control)
[^11^C]DED	MAO-B	Irreversible	No significant difference in AD Increase in Aβ + MCI, pre-symptomatic mutation carrier of ADAD, ALS, CJD, TBI
[^11^C]SL25.1188	MAO-B	Reversible	Increase in major depressive disorder A trend of decrease in PTSD
[^11^C]BU99008	I_2_BS	Reversible	Increase in early PD and Aβ + prodromal AD Decrease in late PD
[^18^F]THK-5351	Tau/MAO-B	Reversible	Increase in AD*, CBS*, PSP, MSA-C, SD, bvFTD, ALS, CJD, TBI, cerebral infraction, progressive multifocal leukoencephalopathy, multiple sclerosis, ischemic stroke, glioma associated with gliosis
[^18^F]SMBT-1	MAO-B	Reversible	Increase in AD

*The uptake may be due to binding to tau aggregates.

### Recent Progress in the Development of Novel Monoamine Oxidase-B Tracers

Although several PET tracers have been evaluated for imaging reactive astrogliosis in humans, most of them were labeled with carbon-11, which limits widespread clinical applications. ^18^F-labeled MAO-B PET tracers such as [^18^F]fluorodeprenyl, [^18^F]fluororasagiline, [^18^F]fluorodeprenyl-D2, and [^18^F]fluororasagiline-D2 have been developed based on MAO-B inhibitors (Nag et al., 2011, 2012a,b, 2013, 2016). However, these tracers possess irreversible binding properties against MAO-B. More recently, development of reversible ^18^F-labeled MAO-B tracers such as [^18^F]FSL25.1188, [^18^F]FBPO, and [^18^F]GHC200449 have been reported (Dahl et al., 2019; Yoshimoto et al., 2019; Nag et al., 2020; Dukic-Stefanovic et al., 2021).

**FIGURE 3:**
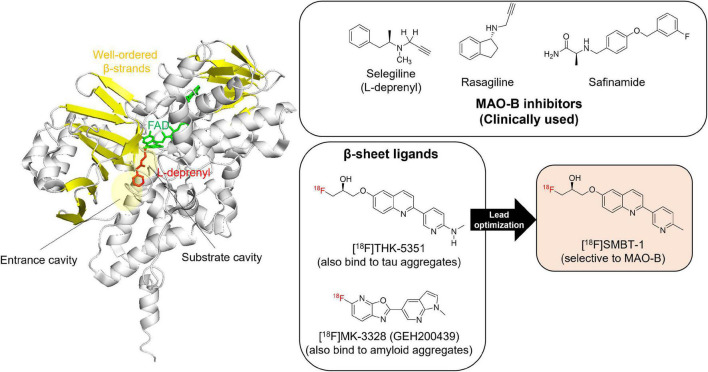
Crystal structure of MAO-B complex with L-deprenyl. Molecular image of 2BYB (De Colibus et al., 2005) created using PyMOL software. Yellow: β-strands. Red: L-derepnyl. Chemical structures of clinically used MAO-B inhibitors (selegiline, rasagiline, and safinamide), β-sheets ligands that binds to MAO-B ([^18^F]THK-5351 and [^18^F]MK-3328), and a novel MAO-B PET tracer, [^18^F]SMBT-1. Although the detailed binding site of β-sheets ligands such as [^18^F]THK-5351 and [^18^F]MK-3328 remains unknown, the evidence suggest that β-sheet ligands are potential MAO-B binders. In fact, lead optimization of one of β-sheet ligands, [^18^F]THK-5351, generated a novel MAO-B PET tracer, [^18^F]SMBT-1.

MAO-B contains β-sheet rich regions that create a substrate cavity space and an entrance cavity space (Figure 3). Irreversible MAO-B inhibitors contain an *N*-propargyl group covalently attached to the *N*5 atom of the FAD cofactor of MAO-B, which is adjacent to β-sheet rich region. *In vitro* competitive binding assays indicated that THK-5351 exhibits competitive binding with both reversible and irreversible MAO-B inhibitors. A docking simulation was reported to understand the binding site of tau PET tracers including THK-5351 in MAO-B (Murugan et al., 2019). Although the detailed binding site of THK-5351 remains unknown, the evidence suggest that β-sheet ligands are potential MAO binders (Figure 3). In fact, validation studies of Aβ and tau PET tracers identified that some of them possess high binding affinity against MAO-B (Sur et al., 2010; Ng et al., 2017; Harada et al., 2018; Vermeiren et al., 2018; Drake et al., 2019). [^18^F]MK-3328 (GEH200439), which was originally developed as a candidate for Aβ PET tracer, showed the interaction with MAO-B in human brains (Sur et al., 2010; Figure 3). However, this tracer showed high non-specific binding in the white matter (Hostetler et al., 2011; Nag et al., 2020).

**FIGURE 4:**
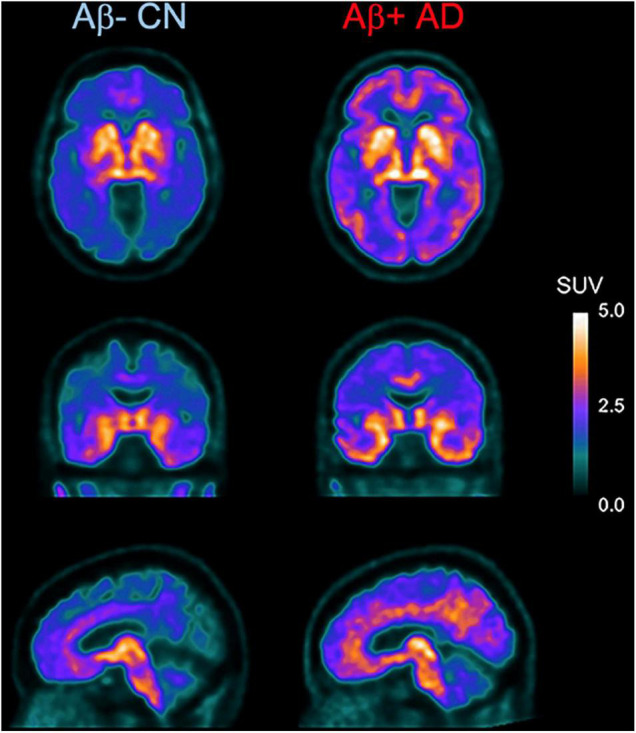
Axial, coronal, and sagittal images showing higher and widespread binding of [^18^F]SMBT-1 in an Aβ-positive Alzheimer’s disease patient (Aβ + AD, right) compared to an age-matched Aβ-negative cognitive unimpaired control (Aβ- CN, left). (Courtesy of Drs. Victor Villemagne and Christopher Rowe).

As mentioned above, [^18^F]THK-5351 PET can detect MAO-B with highly sensitivity. Therefore, we developed a novel reversible and selective MAO-B tracer, [^18^F]SMBT-1 through compound optimization of [^18^F]THK-5351, which is one of the β-sheet ligands. [^18^F]THK-5351 possess a good pharmacokinetic (PK) profile, reflected in high brain uptake and rapid washout without defluorination in humans (Lockhart et al., 2016; Betthauser et al., 2017; Hsiao et al., 2017). Our strategy was to develop compounds that reduce the binding affinity to tau aggregates while preserving the binding affinity to MAO-B and the good PK profile. Previous structure–activity relationship (SAR) analysis of 2-arylquinoline derivatives showed that the 2-amino group on the pyridine ring of 2-arylquinoline derivatives was essential for binding to tau aggregates. SAR analysis of MAO-B using our compound library revealed that the hydroxy group in the 3-fluoro-2-hydroxypropyl (FHP) group of 2-arylquinoline plays an important role in achieving high binding affinity for MAO-B. Therefore, we synthesized 2-arylquinoline derivatives that has FHP group. Lead optimization process revealed that the 2-methylpyridine derivative ([^18^F]SMBT-1) was the best candidate for imaging MAO-B *in vivo* (Harada et al., 2021). Preclinical studies of [^18^F]SMBT-1 demonstrated that high binding affinity and high binding selectivity to MAO-B with low non-specific binding as well as good PK and metabolic profiles (Harada et al., 2021). Preliminary cross-sectional human PET studies in a wide range of ages are indicating that [^18^F]SMBT-1 is a selective MAO-B tracer with low non-specific binding, high entry into the brain while displaying favorable reversible kinetics, and have significantly higher binding in Aβ + AD (Figure 4) and Aβ + cognitively unimpaired controls (Villemagne et al., 2022), similarly to what has been reported with plasma GFAP (Verberk et al., 2020; Chatterjee et al., 2021). These findings suggest that reactive astrogliosis as measured by MAO-B through [^18^F]SMBT-1 is associated with early Aβ accumulation, providing strong support for its use as surrogate marker of astrogliosis. Ongoing clinical studies will also clarify the potential usefulness of [^18^F]SMBT-1 in other disease conditions.

### Imaging of Monoamine Oxidase-B in Animal Models: Implication of Reactive Astrogliosis

Although there are several clinical studies using [^11^C]DED, only several preclinical studies have been reported. Aβ deposition and astrogliosis in APP_Swe_ mice were evaluated using the Aβ PET tracer [^11^C]AZD2184 and [^11^C]DED. Significant [^11^C]DED binding was observed before significant accumulation of Aβ deposits in the brain, although the *in vivo* PET finding was inconsistent with the *in vitro* autoradiography results of [^3^H]-L-deprenyl (Rodriguez-Vieitez et al., 2015). Another preclinical imaging study using [^11^C]DED in APP_ArcSwe_ mice showed a trend of increasing tracer binding in APP_ArcSwe_ mice compared with age-matched wildtype mice, but there was large intragroup variation (Olsen et al., 2018). There was little co-localization between MAO-B and GFAP in these mice, in contrast with good correlation between MAO-B and GFAP in postmortem human brain tissues (Levitt et al., 1982; Jo et al., 2014). This discrepancy could be due to species differences of MAO-B between mice and humans. MAO-B is predominantly expressed in humans and not in rodents. MAO-B expression levels in rodents were 2.5–4.7-fold lower than those in humans (Saura et al., 1992). A head-to-head comparison study of [^11^C]-L-deprenyl, [^11^C]DED, and [^11^C]SL25.1188 in unilateral intrastriatal lipopolysaccharaide (LPS) injected rat model demonstrated that specific binding was only observed in [^11^C]-L-deprenyl (Narayanaswami et al., 2021). They explained the reasons due to faster metabolism of [^11^C]DED in rats and higher non-specific binding and lower binding affinity of [^11^C]SL25.1188 to rat brain tissues. [^18^F]FEPPA, one of the TSPO PET tracers, showed higher binding in the injection site at 1 week post-LPS injection, while [^11^C]-L-deprenyl did not. However, although there were lower uptake of [^18^F]FEPPA at 1-month post-LPS injection than 1-week time point, [^11^C]-L-deprenyl increased the binding in the 2-week and 1-month time points, suggesting increase of MAO-B expression is a later phase of neuroinflammation in this rat model. A study using *in vivo* two-photon microscopy demonstrated the close correlation between the MAO activity and AD progression (Kim et al., 2016). This study demonstrated that MAO activity started to increase in parallel with the deposition of Aβ plaques even in 4 month-old 5 × FAD mice, suggesting that MAO-B expression in reactive astrocytes occur at the early stages of disease in mice.

GFAP-positive reactive astrogliosis were observed not only in *APP* transgenic mice but also in other animal models such as doxycycline inducible astrocytic MAO-B transgenic mice (Mallajosyula et al., 2008), ischemia models (Boutin et al., 2015), LPS-injected animals (Ory et al., 2015), tau transgenic mice (Ramsden et al., 2005), and *APP* knock-in mice (Saito et al., 2014). Development of highly sensitive MAO-B tracers would allow for improved *in vivo* PET imaging in animal models. In addition, further studies are required to find other possible targets for imaging reactive astrogliosis and to develop a technique to discriminate between beneficial and toxic immune responses.

## Conclusion

Reactive astrogliosis occurs commonly not only in AD but also in various neurological conditions. *In vivo* imaging of reactive astrogliosis is potentially useful for early and differential diagnosis, assessment of disease severity and evaluating drug efficacy. Several promising PET tracers for imaging reactive astrogliosis have emerged. Further clinical evaluation and preclinical validations are required to better understand the pathophysiological role(s) of reactive astrogliosis in neurodegeneration and to develop new treatment strategies.

## Author Contributions

RH and NO wrote the manuscript. SF, YK, KY, and VV revised the manuscript. All authors contributed to the article and approved the submitted version.

## Conflict of Interest

NO and YK hold stocks in CLINO Co., Ltd. RH, SF, and NO were scientific consultants for CLINO Co., Ltd. The authors declare that this study received funding from Clino Ltd. The funder was not involved in the study design, collection, analysis, interpretation of data, the writing of this article, or the decision to submit it for publication.

## Publisher’s Note

All claims expressed in this article are solely those of the authors and do not necessarily represent those of their affiliated organizations, or those of the publisher, the editors and the reviewers. Any product that may be evaluated in this article, or claim that may be made by its manufacturer, is not guaranteed or endorsed by the publisher.
